# Classification of Myelodysplastic, Myeloproliferative, and Myelodysplastic/Myeloproliferative Neoplasms: The Past, Present, and Future

**DOI:** 10.1002/ajh.27656

**Published:** 2025-03-08

**Authors:** Daniel A. Arber, Attilio Orazi

**Affiliations:** ^1^ Department of Pathology University of Chicago Chicago Illinois USA; ^2^ Department of Pathology Texas Tech University Health Sciences Center El Paso El Paso Texas USA

**Keywords:** classification, myelodysplastic/myeloproliferative neoplasms, myelodysplastic syndrome, myeloproliferative neoplasms

## Abstract

With the recent publication of new classification systems of hematopoietic neoplasms, understanding how recognition of disease entities has occurred over time and the subsequent development of formal disease classifications is of importance. This review focuses on the early recognition of myeloid disorders, especially chronic myeloid disorders, and how clinical observations became associated with specific cytologic, histologic, immunophenotypic, and eventually genetic features. This combined approach to disease classification is of particular importance in the evaluation of chronic myeloid neoplasms and has resulted in the definition of clinicopathologic disease entities that allow for more customized treatment approaches. The constant incorporation of ever‐increasing information related to these disorders illustrates that disease classification is a constantly evolving process that requires constant updates as we strive to better understand the disorders we diagnose and treat.

## 1 Introduction and Historic Discoveries of Myeloid Neoplasms

The classification of myeloid neoplasms has evolved over many decades, if not centuries, partly due to emerging technologies and medical specialization in pathology and hematology. The history of these disorders is well‐described [1, 2, 3, 4], and will only be briefly reviewed here. The humors that were felt to influence medical conditions in the times of Hippocrates and Galen were based on gross features of blood, including serum (yellow bile), white blood cells (phlegm) and red blood cells (blood), and clotted blood or cold and dry substances thought to be secreted from the spleen (black bile). Despite the concept of phlegm being associated with disease, most commonly infections, the first clinical reports of probable chronic myeloid neoplasms did not occur until 1811 when Cullen [5] reported a case of “splenitis acutus” in which the patient's serum was described as milk like, presumably due to a markedly elevated white blood cell count. This was followed by similar reports by others over the next few years [6, 7, 8, 9]. These reports, however, were based on observation of bloodletting samples, as well as clinical and postmortem features of patients presumably with acute or chronic myeloid neoplasms. Although the scientific use of the microscope began in the late 17th century, it was approximately 150 years later that it was used in medicine [10, 11]. Donné published what is considered the first cytologic description of blood using the microscope in 1839. In 1845, several reports of the microscopic description of blood disorders were published with Bennett introducing the term “leucocythemia” in what was probably the first report of microscopic features of blood in a patient with chronic myeloid leukemia (CML) [12, 13]. That same year, Virchow, considered by many as “the father of pathology,” introduced the term “weisses blut” for white blood cells due to their microscopic appearance on unstained smears [14]. He later introduced the term “leukämie” for proliferations of those cells [15]. Virchow also proposed that these leukemias, presumably acute and chronic, could be divided based on splenic or lymphatic presentation [15].

Despite these advances in the uses of the microscope in medicine, it was not until 1846 that Fuller and Cantab diagnosed a leukemic proliferation in a living patient using microscopic examination of peripheral blood [16]. In 1869, Neumann finally discovered a link between the bone marrow and these disorders [17]. This discovery opened the door to a better understanding of the complexity of the diseases previously viewed as “leukemia.” While the concepts of acute and chronic remained to be clarified, the idea that different disorders arose in the bone marrow versus the spleen and lymph nodes was a critical finding.

The next major discovery to occur was in the work of Ehrlich in 1877 when he began to apply new discoveries in aniline dyes to stain peripheral blood and other smears to allow for more detailed examination of the cells that were previously simply called white blood cells [18]. The ability to observe specific staining patterns of blood and bone marrow cells became critical to the discovery of specific cell types involved in myeloid disorders.

With these tools, clinicians began to identify differing clinical and hematologic features of patients, and based on case reports and small series, early disease entities were described. What we now consider primary myelofibrosis was described in several reports in what was termed *osteosclerotic anemia* or *Heuck‐Assmann syndrome* [19, 20, 21, 22, 23, 24]. William Osler, summarizing his own cases [25] and prior case reports [26, 27, 28, 29, 30] proposed what is now considered polycythemia vera as a clinical entity in 1903. This was followed by the description of di Guglielmo's syndrome in 1917 [31] and essential thrombocythemia in 1934 [32]. By the early 20th century, four main types of leukemia were classified: chronic lymphocytic leukemia (CLL), chronic myelogenous leukemia (CML), acute lymphocytic leukemia (ALL), and erythroleukemia.

What was missing from these descriptions, however, were strict diagnostic criteria and an understanding of a possible interrelation between these entities that we now know have overlapping clinical presentations. The latter was addressed, in part, by the proposal of Dameshek in 1951 of the unifying term of myeloproliferative disorders [33], that brought together at least some of the chronic myeloid neoplasms into a common group, suggesting that there were all “manifestations of proliferative activity of marrow cells due to a hitherto undiscovered stimulus.” Dameshek described differentiating bone marrow and extramedullary features of various cell types for chronic granulocytic leukemia, polycythemia vera, idiopathic or agnogenic myeloid metaplasia of the spleen, megakaryocytic leukemia, and erythroleukemia (including di Guglielmo syndrome) in what might be considered an early classification of myeloproliferative neoplasms (MPNs).

## 2 Evolving Classifications

The second half of the 20th century marked the emergence of multiple classifications that included chronic myeloid disorders. While Henry Rappaport's classification of malignant lymphomas began in the late 1950s, his 1966 Armed Forces Institute of Pathology monograph entitled “Tumors of the Hematopoietic System” [34] went beyond malignant lymphoma and included myeloid neoplasms, including a category of myeloproliferative diseases and an entity of chronic monocytic leukemia. While well illustrated, the monograph did not provide specific diagnostic criteria for the entities discussed.

In 1971, the Polycythemia Vera Study Group (PVSG) [35] published its approach to the diagnosis of polycythemia vera based on red‐cell mass, arterial oxygen saturation, presence or absence of splenomegaly, platelet and white blood cell counts, leukocyte alkaline phosphatase score, and serum B_12_ levels. The diagnostic criteria did not include peripheral blood or bone marrow morphologic features, and a bone marrow biopsy was not required for diagnosis. The group went on to propose diagnostic criteria for essential thrombocythemia [36].

In 1976, the World Health Organization (WHO), in the first edition series of the International Histological Classification of Tumors, published “Histological and Cytological Typing of Neoplastic Diseases of Haematopoietic and Lymphoid Tissues” by Mathé and Rappaport [37] which, in many ways, was an abbreviated form of Rappaport's prior classification. It included a broad category of CML and other myeloproliferative diseases as well as chronic monocytoid (monocytic) leukemia but again, there were no real diagnostic criteria, and the classification was not widely adopted. The second edition tumor series from the WHO did not include a monograph on hematopoietic neoplasms.

Of more significance, 1976 marked the publication of the French‐American‐British Cooperative Leukemia Group (FAB) [38] that provided detailed diagnostic criteria for acute leukemia, followed in subsequent years by the publication of criteria for a number of disorders, including myelodysplastic syndromes (MDS) and other chronic myeloid disorders [39, 40]. The FAB elevated what was called up to that time preleukemia to myelodysplastic syndrome with specific criteria on blast percentages, establishing a variety of disease categories ranging from *refractory anemia* to *refractory anemia with excess blasts in transformation*. The FAB also included chronic myelomonocytic leukemia (CMML) as a type of MDS. In 1994, the same group described atypical CML, providing criteria for differentiating it from chronic granulocytic leukemia (now known as CML) and CMML [40].

For some of these disease entities, bone marrow aspirate smear criteria were proposed. It is of note that other than the contributions of Rappaport, which were more in collating existing data on myeloid disorders, the key advances in the discovery and classification of myeloid disorders up until the late 1980s were the result of efforts of hematologists and, in terms of morphology, were focused primarily on peripheral blood and aspirate smear cytology.

Bone marrow trephine biopsy histology began to be introduced in more formal classification proposals in the 1980s, especially in the area of chronic myeloproliferative disorders [41, 42]. The so‐called Hannover Classification [42] described different morphologic patterns found on trephine biopsies in CML, and subsequent studies identified distinctive histologic features that distinguished prefibrotic primary myelofibrosis (pre‐PMF) from essential thrombocythemia and polycythemia vera [43].

**FIGURE 1:**
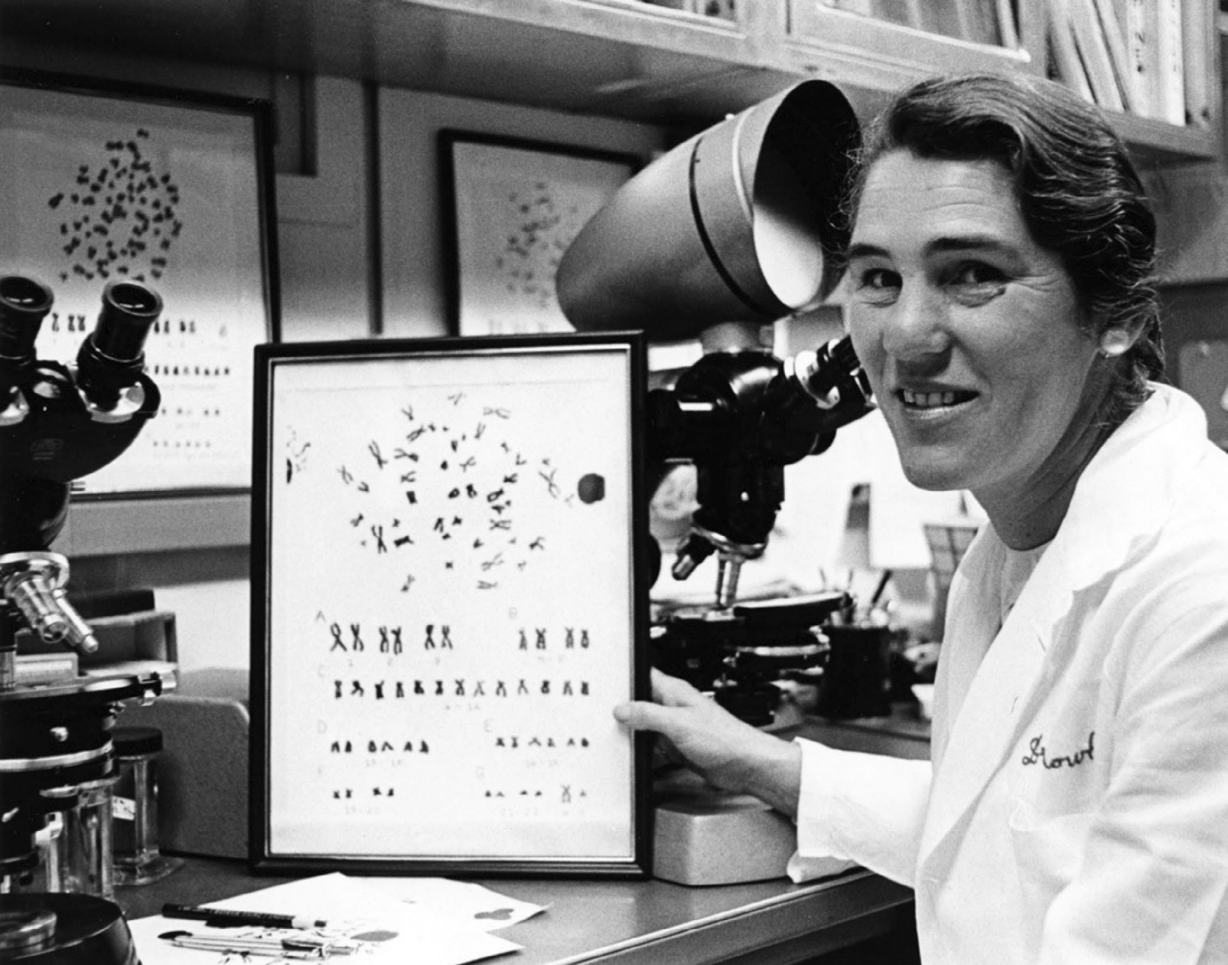
Dr. Janet Rowley characterized the “Philadelphia chromosome” of chronic myeloid leukemia to represent a balanced cytogenetic translocation between chromosomes 9 and 22. She later discovered numerous other cytogenetic abnormalities in myeloid disorders and successfully moved karyotype analysis from the research laboratory to the clinical practice. This work began the modern era of molecular diagnostics in myeloid neoplasms. University of Chicago Photographic Archive, apf1‐12 883, Hanna Holborn Gray Special Collections Research Center, University of Chicago Library.

Similar to the delay in using microscopy in the diagnoses and classification of myeloid disorders in the 17th and 18th centuries, there was a similar delay in recognizing the importance of genetic abnormalities in defining these disorders as well. Of course, the techniques for genetic discoveries were initially crude and advanced relatively slowly in the 20th century, Nowell and Hungerford identified the Philadelphia chromosome and its association with CML in 1960 [44]. By 1973, Janet Rowley (Figure 1) characterized the Philadelphia chromosome as representing a balanced translocation of chromosomes 9 and 22 [45], and the era of diagnostic cytogenetics had begun. The Hannover Classification merged morphology with genetics by recognizing morphologic variants of CML that mimicked other MPNs but carried the *t*(9;22).

## 3 The Evolution of Multiparameter Pathologic Classifications

While the focus of this review is myeloid neoplasms, the modern era of hematopathology classification began with the Revised European American Lymphoma (REAL) Classification published in 1994 [46]. This lymphoma classification was proposed by the International Lymphoma Study Group (ILSG), a group of expert hematopathologists, and defined disease entities based on a combination of clinical features, morphology, immunophenotyping, and genetic studies. The group approach to the classification helped it gain acceptance in a world of competing lymphoma classifications but was initially criticized for lacking input from treating physicians and a broader group of pathologists [47]. To address these concerns, the classification was validated to demonstrate prognostic significance and clinical utility with active clinical and broader pathologist input [48, 49]. This new approach to classification interested leaders of the 3rd edition WHO classification, and they approached Elaine Jaffe, an author of the REAL classification and, at that time, the president of the Society for Hematopathology, and asked her and the society to lead an effort to use a similar approach for the WHO 3rd edition classification of tumors of the hematopoietic and lymphoid tissues. Jaffe and the Society for Hematopathology agreed to partner with the WHO in this effort under the conditions that the European Association for Haematopathology also be included and that the classification be developed in conjunction with a Clinical Advisory Committee meeting (CAC) to ensure there was sufficient input from a broad community of international pathologists, geneticists, hematologists, and oncologists prior to publication. The CAC was organized by the pathology societies, independent of the WHO but with WHO support, and the findings were published in peer‐reviewed journals [50] in advance of the full book publication by the International Agency for Research on Cancer (IARC)/WHO [51].

The 3rd edition WHO classification, published in book form in 2001 [51], resulted in a comprehensive and widely accepted classification of lymphoma and myeloid neoplasms. Similar approaches were successfully employed for the 4th (200) [52] and revised 4th (2017) [53] editions of the WHO classification, and these classifications were regarded as the standard for disease classification of hematopoietic tumors. Unfortunately, new leadership in the oversight of disease classification for the 5th edition WHO series ended the partnership with the hematopathology societies and support for the CAC process for informing classification changes. The hematopathology societies chose to continue the prior CAC process, which resulted in the International Consensus Classification of Myeloid and Lymphoid Neoplasms [54, 55, 56] while the WHO published drafts and eventually a final version of a 5th edition classification [57, 58, 59]. Because both classifications were informed by the same literature, they have many similarities, but some differences exist. The following sections will highlight many of these differences but should not be considered exhaustive.

## 4 Myelodysplastic Syndromes

Table 1 summarizes key changes in the classification of MDS from the FAB through the current classifications. The FAB and earlier WHO classifications retained the term *refractory anemia* for most categories, despite the knowledge that these disorders were neoplastic. The revised 4th edition WHO classification changed the terminology to *myelodysplastic syndrome* in the specific entity names to better reflect the specificity of the diagnosis in contrast to the vaguer *refractory anemia* terminology. The WHO 5th edition changes the name of the entire category to *Myelodysplastic Neoplasms*, although they retain the abbreviation of MDS for this group. While these disorders are accepted as neoplasms, such a change in terminology was discussed and rejected by the Clinical Advisory Committees of the revised 4th edition WHO classification and the ICC, and both retained the category name *Myelodysplastic Syndromes*. Disease categories have been added and deleted from this category over time. CMML, originally considered an MDS by the FAB classification, was moved to a new disease group of myelodysplastic/myeloproliferative diseases by the WHO 3rd edition (the category remains, but the name was changed to myelodysplastic/MPNs in subsequent classifications). Of particular note, the WHO added categories of *refractory cytopenia with multilineage dysplasia* and *MDS associated with isolated del(5q)* (3rd edition) and added a provisional category of *refractory cytopenia of childhood* (4th edition). The later provisional entity is retained but was moved to the category of *Pediatric and/or Germline Mutation‐Associated Disorders* in the ICC, recognizing that not all cases are clearly neoplastic. In contrast, the WHO 5th edition eliminated the prior terminology and appears to include such cases in a new entity of *childhood myelodysplastic neoplasm with low blasts*, making it more difficult to further study this unusual group of disorders [60].

**TABLE 1:** Major classification milestones in myelodysplastic syndromes.

FAB	WHO 3rd edition	WHO 4th edition	WHO revised 4th edition	WHO 5th edition	ICC
Disease groups Refractory anemia (RA) RA with ring sideroblasts (RARS) RA with excess blasts (RAEB) Chronic myelomonocytic leukemia (CMML) RAEB in transformation (RAEB‐T)	Added Refractory cytopenia with multilineage dysplasia Myelodysplastic syndrome (MDS), unclassifiable MDS associated with isolated del(5q) chromosome abnormality Moved • CMML to new category of myelodysplastic/myeloproliferative disease	Changed RA to refractory cytopenia with unilineage dysplasia Added Childhood myelodysplastic syndrome with provisional entity of *refractory cytopenia of childhood*	Nomenclature Changed *RA* to *MDS* (myelodysplastic syndrome) in entity names Changed RA with unilineage dysplasia to RA with single lineage dysplasia	Nomenclature Changed disease category name to *Myelodysplastic Neoplasms* Change MDS with ring sideroblasts to myelodysplastic neoplasm with low blasts and *SF3B1* mutation Introduce low blasts (MDS‐LB) and increased blasts (MDS‐IB1 and MDS‐IB2) and increased blasts with fibrosis (MDS‐F) into diagnostic category names Added Myelodysplastic neoplasm with biallelic *TP53* inactivation Myelodysplastic neoplasm, hypoplastic Childhood myelodysplastic neoplasm with low blasts Childhood myelodysplastic neoplasm with increased blasts Eliminated Unclassified category	Nomenclature Nongenetic groups without a blast increase changed to MDS, NOS categories Added Genetic categories based on *SF3B1* and *TP53* mutations MDS/AML to replace prior MDS‐EB2 in adults
Diagnostic criteria Blast criteria established to differentiate disease groups from each other and from acute myeloid leukemia (defined as ≥ 30% blasts)	Diagnostic criteria Eliminated RAEB‐T, lowering the blast cell cutoff for AML to > 20%	Diagnostic criteria No major change	Diagnostic criteria Lowered ring sideroblasts requirement for MDS with ring sideroblasts to 5% or more if *SF3B1* mutation is present Provided specific criteria for MDS, unclassified	Diagnostic criteria No major change	Diagnostic criteria Require VAF ≥ 10% for *SF3B1* and *TP53* mutations Require multi‐hit *TP53* mutations for cases with < 10% peripheral blood or bone marrow blasts

**FIGURE 2:**
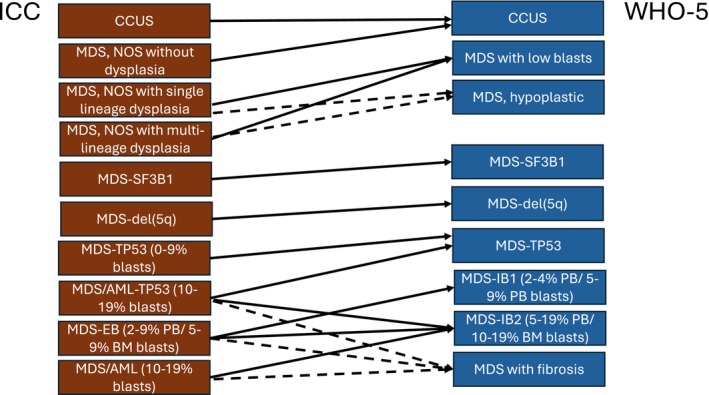
Comparison of ICC myelodysplastic syndrome (MDS) groups to the WHO 5th edition (WHO‐5) myelodysplastic neoplasms (MDS) groups. Cases of MDS‐SF3B1 (WHO‐5) may be diagnosed with 15% or more ring sideroblasts in the absence of an *SF3B1* mutation in the WHO‐5 while such cases would be classified in the MDS, NOS category of the ICC. MDS‐TP53 in both classifications require multi‐hit *TP53* mutations, but the criteria for multi‐hit vary slightly and the ICC requires a variant allele frequency of 10% or more. Cases of MDS/AML‐TP53 (ICC) would be classified as MDS‐TP53 (WHO‐5) if multi‐hit, but as MDS‐IB2 if single hit. CCUS, clonal cytopenia of undetermined significance; MDS, NOS, MDS, not otherwise specified; MDS/AML‐TP53, MDS/acute myeloid leukemia with mutated *TP53* (ICC); MDS‐del(5q), MDS with del(5q) (ICC) and MDS with low blasts and 5q deletion (WHO‐5); MDS‐EB, MDS with excess blasts (ICC); MDS‐IB1, MDS with increased blasts‐1 (WHO‐5); MDS‐IB2, MDS with increased blasts‐2 (WHO‐5); MDS‐SF3B1, MDS with mutated *SF3B1* (ICC) and MDS with low blasts and *SF3B1* mutation (WHO‐5); MDS‐TP53, MDS with mutated *TP53* (ICC) and MDS with biallelic *TP53* inactivation (WHO‐5).

Both new classifications (Figure 2) now include MDS categories defined by the presence of gene mutations. Both recognize MDS with mutated *SF3B1* as an entity, typically associated with ring sideroblasts, and lacking an increase in peripheral blood or bone marrow blasts. The ICC requires a variant allele frequency (VAF) of 10% or more for such a diagnosis. Both also recognize the importance of *TP53* mutations in MDS. Both require multi‐hit or biallelic mutations of *TP53* for cases with less than 10% blast cells. The WHO 5th edition also requires biallelic *TP53* mutations for cases with higher blast counts (10%–19%) (all termed MDS with biallelic *TP53* inactivation), while the ICC allows cases with 10%–19% blasts to be diagnosed as a new category of MDS/AML with mutated *TP53* based on the presence of a single mutation. The ICC requires a VAF of ≥ 10% for single and multi‐hit *TP53* mutations; the WHO has no VAF criteria for diagnosis. The new ICC category of MDS/AML is not limited to cases with *TP53* mutations and, in adults, replaces the prior category of MDS with excess blasts‐2. Pediatric cases with blasts between 10% and 19% do not fall into the new MDS/AML category and would be diagnosed as MDS with excess blasts in the ICC. In addition, the WHO formally recognized both MDS with a hypoplastic marrow (MDS, hypoplastic) and MDS with fibrosis. Due to its blast number variability, the former is considered on its own as a separate entity, while the latter is a subentity within the spectrum of MDS with increased blasts (MDS‐IB).

While disease classifications are important to pathologists as well as treating physicians, other factors that are not included in pathologic classifications often aid in the prediction of prognosis in hematologic disorders. Because of this, many prognostic scoring systems have been developed and evolved over time. For MDS, the International Prognostic Scoring System (IPSS) [61] and its revision (IPSS‐R) [62] incorporated other features, including the number and degree of cytopenias and more detailed blast cell categories, as well as defining cytogenetic risk groups. Using these parameters, scoring systems were developed that identified prognostic groups. This approach was expanded in the IPSS‐M [63] which assesses the prognostic importance of 36 gene mutations. This gene panel includes *SF3B1* and *TP53*, gene mutations captured by the current MDS classifications, but includes many other genes important for prognostic determination in these disorders.

## 5 Myeloproliferative Neoplasms

As mentioned, the discovery of the Philadelphia chromosome and its characterization started a modern era of discovery, especially in the MPNs. These discoveries ultimately led to the introduction of tyrosine kinase inhibitors. While the morphologic features of most of the MPNs were well‐described, the introduction of genetic markers of disease, including the associations with *JAK2*, *MPL*, and *CALR* mutations, greatly aided in the differential diagnosis of these disorders. Similarly, the discovery of *CSF3R* mutations in chronic neutrophilic leukemia greatly enhanced pathologists' ability to diagnose that disorder. In the presence of a *CSF3R* mutation, the ICC lowered the WBC requirement to > 13 × 10^9^/L, down from the prior > 25 × 10^9^/L, while the WHO 5th edition still maintains the prior WBC threshold.

**FIGURE 3:**
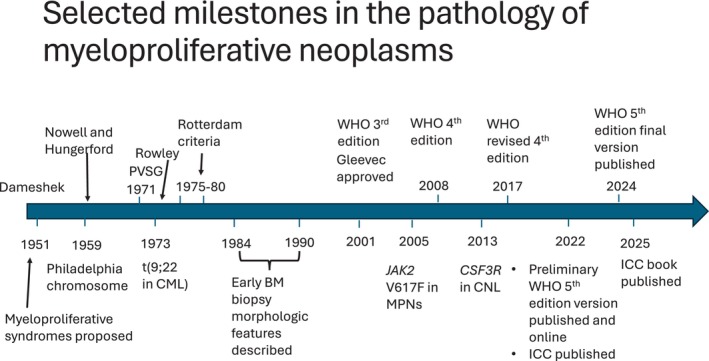
Selected milestones in the pathology of myeloproliferative neoplasms. BM, bone marrow; CML, chronic myeloid leukemia; CNL, chronic neutrophilic leukemia; ICC, International Consensus Classification; MPNs, myeloproliferative neoplasms; PVSG, Polycythemia Vera Study Group; WHO, World Health Organization.

Figure 3 outlines some of the major discoveries related to MPNs. One of the noteworthy advances is in the characterization of early/prefibrotic primary myelofibrosis [43]. Of note, before the WHO 3rd edition, primary myelofibrosis was termed chronic idiopathic myelofibrosis, and pre‐PMF was not recognized, being often confused with essential thrombocythemia. Recognition of this early form of PMF allows for better disease prognostication. Another key advance has been in the identification of a group of genetically distinct disorders that have variable pathologic presentations but are commonly associated with eosinophilia. The category of *Myeloid and Lymphoid Neoplasms with Eosinophilia and Abnormalities of PDGFA*, *PDGFRB*, and *FGFR1* was introduced in the WHO 4th edition and continues to grow with more associated genetic abnormalities included in subsequent classifications [64]. This category provides a genetic link to a given disease in spite of differing morphologic and immunophenotypic manifestations in coexisting or subsequent specimens. In the past, these disease manifestations would have been considered unrelated, that is, produced by different conditions.

Despite many similarities between the ICC and the WHO 5th edition in terms of MPNs, there are a few differences. The WHO 5th edition now includes juvenile myelomonocytic leukemia (JMML) as an MPN. JMML was previously considered a myelodysplastic/MPN in the prior WHO classifications. The ICC has moved this disorder to the category of *Pediatric and/or Germline Mutation‐Associated Disorders* based on its genetic profile and its similarity to other disorders in this category and has introduced a new category of JMML‐like neoplasms [60, 65].

Also of significance, and controversy [66, 67, 68, 69], is that the WHO 5th edition eliminates the accelerated phase of CML arguing that “high‐risk features” should be considered rather than the historic definition of accelerated phase in CML. Of note, the WHO 5th edition retains the accelerated phase for other MPNs. The ICC retains the accelerated phase in CML, defined as bone marrow or peripheral blood blasts of 10%–19%, peripheral blood basophils of 20% or more, or the presence of additional clonal cytogenetic abnormalities in neoplastic (*BCR::ABL1* positive) cells [70].

Prognostic scoring systems for MPNs do not include specific morphologic features and initially included age, specific blood count features, including blast cell increases, as well as the presence or absence of constitutional symptoms [71, 72]. Over time, revised and new scoring systems have added karyotype findings [73, 74] and molecular genetic findings [74, 75]. The addition of gene mutation studies, combined with other findings, has identified high‐risk or very high‐risk disease groups for PMF (presence of *ASXL1*, *SRSF2* or absence of *CALR* type 1 mutations), ET (*SRSF2*, *SF3B1*, *U2AF1*, and *TP53* mutated), and PV (*SRSF2* mutated) that cannot be predicted by the current pathologic classification of these disorders.

## 6 Myelodysplastic/Myeloproliferative Neoplasms

**FIGURE 4:**
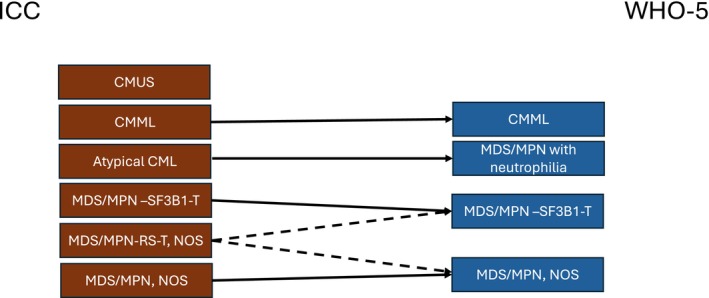
Comparison of ICC myelodysplastic/myeloproliferative neoplasm (MDS/MPN) groups to WHO‐5 groups. CMUS is not specifically defined by the WHO‐5. There is no distinct category for MDS/MPN‐RS‐T, NOS (lacking mutation of *SF3B1*) in the WHO‐5 and such cases could fall into the MDS/MPN, NOS (WHO‐5) category. Atypical CML, atypical chronic myeloid leukemia (ICC); CMML, chronic myelomonocytic leukemia; CMUS, clonal monocytosis of undetermined significance; MDS/MPN, NOS, MDS/MPN, not otherwise specified; MDS/MPN‐RS‐T, NOS, MDS/MPN with ring sideroblasts and thrombocytosis; MDS/MPN‐SF3B1‐T, MDS/MPN with *SF3B1* mutation and thrombocytosis.

The category of myelodysplastic/myeloproliferative neoplasms (MDS/MPN; termed myelodysplastic/MPNs in the WHO 3rd edition) includes disorders that typically present with both cytosis/cytoses and cytopenia(s), the most common of which is CMML. Key changes over time in this category are summarized in Table 2, and a comparison between the ICC and WHO 5th edition is provided in Figure 4. While the diagnostic criteria for CMML have evolved across classifications, such as the allowance for myeloid associated gene mutations as a marker of clonality, the primary criteria have not significantly changed until the current classifications. Both now allow for a diagnosis of CMML with a peripheral blood absolute monocyte count of less than 1.0 × 10^9^/L, lowering the threshold to 0.5 × 10^9^/L when at least 10% of the WBC are monocytes and other criteria are met. This change is supported by the finding that such cases have a high rate of progression to more typical CMML [76, 77, 78]. The ICC and WHO 5th edition, however, differ in that the ICC requires the detection of a clonal cytogenetic or molecular genetic abnormality to be present for cases with less than 1.0 × 10^9^/L monocytes. Other differences specifically required only by the ICC diagnostic criteria include the presence of cytopenia and a supportive marrow morphology [70].

**TABLE 2:** Evolution of MDS/MPN disease groups since the WHO 3rd edition (ed).

WHO 3rd ed	WHO 4th ed	WHO revised 4th ed	WHO 5th ed	ICC
CMML	Moved cases of CMML with eosinophilia and *PDGFRB* rearrangements to new category of *myeloid and lymphoid neoplasms with eosinophilia and abnormalities of* PDGFRA, PDGFRB, *or* FGFR1	Introduced CMML‐0 for cases with < 2% PB and < 5% BM blasts and no Auer rods Introduced MDS and MPN types based on WBC count below and above 13 × 10^9^/L	Eliminated CMML‐0, encompassing such cases in CMML‐1 Lowered absolute monocyte count to > 0.5 × 10^9^/L	Eliminated CMML‐0, encompassing such cases in CMML‐1 Lowered absolute monocyte count to > 0.5 × 10^9^/L, only with proof of clonality
aCML	No major change	No major change	Renamed as *MDS/MPN with neutrophilia*	No major change
JMML	No major change	No major change	Moved to become a category of *myeloproliferative neoplasms*	Moved to become a category of *pediatric and/or germline mutation‐associated disorders* and requires mutations in canonical RAS pathway genes Introduced new category of *JMML‐like neoplasms*
		Added new category of *MDS/MPN with ring sideroblasts and thrombocytosis*	Renamed as *MDS/MPN with SF3B1 mutation and thrombocytosis*	Renamed as *MDS/MPN with thrombocytosis and* SF3B1 *mutation* or *MDS/MPN with ring sideroblasts and thrombocytosis, not otherwise specified*
MDS/MPN, U	No major change	No major change	Name changed to *MDS/MPN, NOS (U)*	Name changes to *MDS/MPN, NOS*

Abbreviations: aCML, atypical chronic myeloid leukemia; BM, bone marrow; CMML, chronic myelomonocytic leukemia; ICC, International Consensus Classification; JMML, juvenile myelomonocytic leukemia; MDS/MPN, myelodysplastic/myeloproliferative neoplasm; NOS, not otherwise specified; PB, peripheral blood; U, unclassified; WBC, white blood cell count; WHO, World Health Organization.

The diagnostic criteria for atypical CML (aCML) have not changed significantly with the notable exception that the ICC requires the absence of hypereosinophilia, but the WHO 5th edition has changed the name of this disorder to *myelodysplastic/myeloproliferative neoplasm with neutrophilia*. While aCML represents a well‐established recognizable entity, it is unclear why a disease definition based on the presence of “neutrophilia and dysplasia” would represent progress as the same features are also observed in a high proportion of cases of MDS/MPN, NOS as well as in MPN.

The entity of MDS/MPN with ring sideroblasts and thrombocytosis, first included in the WHO revised 4th edition, is a prototypic example of an overlapping genetic category with frequent gene mutations associated with MPNs (usually *JAK2*) and MDS (*SF3B1*). This disease is now considered a specific, genetically defined entity in the new classifications; termed *MDS/MPN with SF3B1 mutation and thrombocytosis*. However, the ICC also recognizes *MDS/MPN with ring sideroblasts and thrombocytosis*, *not otherwise specified* for cases sharing similar features but lacking a mutation in *SF3B1*. Only the ICC formally recognizes MDS/MPN with isolated isochromosome (17q) [79] that is included as a new provisional subentity under the category of MDS/MPN, NOS.

Prognostic scoring systems for CMML are also provided to aid in survival and acute myeloid leukemia evolution prediction. The CMML‐specific prognostic scoring system (CPSS) [80] includes CMML subtypes defined by past and current pathologic classifications, including CMML‐1 versus CMML‐2, and myeloproliferative versus myelodysplastic subtypes. A scoring system that includes mutation studies (*ASXL1* and *NRAS*) was more recently proposed to predict survival in CMML after transplantation [81].

## 7 Future Directions

As this review implies, the classification of chronic myeloid disorders is constantly impacted by new information that aids us in the diagnosis and prognostication of the various disorders. Our knowledge, particularly on the molecular pathology of these disorders, is growing rapidly with the advent of new investigational tools. Specific genetic abnormalities are disease‐defining in some areas, such as MDS and CML, while they are less specific but confirmatory in others, such as the *BCR::ABL1*‐negative MPNs and the MDS/MPNs. Mutations in *TP53* in MDS (and acute myeloid leukemia) appear to be more important for prognosis than blast cell counts, suggesting that a more molecularly defined approach to these disorders will continue. The boundaries between clonal hematopoiesis and MDS still need better definition, and studies to determine appropriate VAFs to distinguish the two remain in progress. Among the MPNs, early studies suggest mutations in *TP53* may have more prognostic significance than morphology and blast counts, and the role of this gene in MPN progression is an emerging area of investigation [82]. We are only beginning to understand the interplay between gene mutations and epigenetic changes in myeloid disorders, and such combinations may better clarify our ability to accurately diagnose in the future.

As we have learned, however, the diagnosis of myeloid disorders is complicated and relies heavily on a combination of clinical features, morphology, immunophenotyping, and genetics. Understanding this was the cornerstone of the early WHO classifications. Future classifications must continue to be developed as a partnership between pathologists, geneticists, and hematologists using a process similar to the Clinical Advisory Committees that informed the WHO 3rd, 4th, and revised 4th editions and the ICC. This approach allows for careful evaluation of all aspects of diagnosis with broad‐based input and acceptance of any classification.

## Ethics Statement

The authors have nothing to report.

## Consent

The authors have nothing to report.

## Conflicts of Interest

The authors declare no conflicts of interest.

## Data Availability

Data sharing not applicable to this article as no datasets were generated or analyzed during the current study.
